# Supplementary material for: Astronomy’s climate emissions: Global travel to scientific meetings in 2019
Source: PNAS Nexus. 2024 Apr 30;3(5):pgae143. doi: 10.1093/pnasnexus/pgae143 (PMC11060108; doi:10.1093/pnasnexus/pgae143)
Supplement: pgae143_Supplementary_Data [file pgae143_supplementary_data.pdf]

PNAS nexus

Astronomy's climate emissions: global travel to scientific meetings in 2019 – Supplementary Material

Andrea Gokus,[1,2,3*] Knud Jahnke,[4] Paul M. Woods,[5] Vanessa A. Moss,[6,7]
Volker Ossenkopf-Okada,[8] Elena Sacchi,[9] Adam R. H. Stevens,[10]
Leonard Burtscher,[11] Cenk Kayhan,[12] Hannah Dalgleish,[13,14] Victoria Grinberg,[15]
Travis A. Rector,[16] Jan Rybizki,[17] and Jacob White[18]

[1]Department of Physics & McDonnell Center for the Space Sciences,
Washington University in St. Louis, One Brookings Drive, St. Louis, MO 63130, USA

[2]Dr. Karl Remeis Sternwarte & ECAP,
Universität Erlangen-Nürnberg, Sternwartstr. 7, 96049 Bamberg, Germany

[3]Institut für Theoretische Physik und Astrophysik,
Universität Würzburg, Emil-Fischer-Straße 31, 97074 Würzburg, Germany

[4]Max-Planck-Institut für Astronomie, Königstuhl 17, 69117 Heidelberg, Germany

[5]Independent researcher, London, UK

[6]ATNF, CSIRO Space and Astronomy, PO Box 76, Epping, NSW 1710, Australia

[7]Sydney Institute for Astronomy, School of Physics A28, University of Sydney, NSW 2006, Australia

[8]Universität zu Köln, I. Physikalisches Institut, Zülpicher Str. 77, 50937 Köln, Germany

[9]Leibniz-Institut für Astrophysik Potsdam (AIP), An der Sternwarte 16, 14482 Potsdam, Germany

[10]International Centre for Radio Astronomy Research,
The University of Western Australia, Crawley, WA 6009, Australia

[11]Leiden Observatory, PO Box 9513, 2300 RA, Leiden, The Netherlands

[12]Department of Astronomy and Space Sciences,
Faculty of Science, Erciyes University, 38030 Melikgazi, Kayseri, Türkiye

[13]Department of Physics, University of Oxford, Keble Rd, Oxford, OX1 3RH, UK

[14]Politics & International Relations, University of Southampton, University Rd, Southampton, SO17 1BJ, UK

[15]European Space Agency (ESA), European Space Research and Technology Centre (ESTEC)
Keplerlaan 1, 2201 AZ Noordwijk, The Netherlands

[16]University of Alaska Anchorage, 3211 Providence Dr., Anchorage, AK, 99508 USA

[17]Weimar Institute of Applied Construction Research, Über der Nonnenwiese 1, 99428 Weimar, Germany

[18]Independent researcher, Vancouver, Canada

*To whom correspondence should be addressed; E-mail: gokus@wustl.edu / dr.andrea.gokus@gmail.com

Hotel CO_2e emissions

In-person conferences require travel to the conference venue, and also — if not at a local venue — hotel accommodation. Staying at a hotel comes with its own environmental footprint, due to electricity consumption, heating and cooling, catering, as well as waste and wastewater disposal needs. To assess

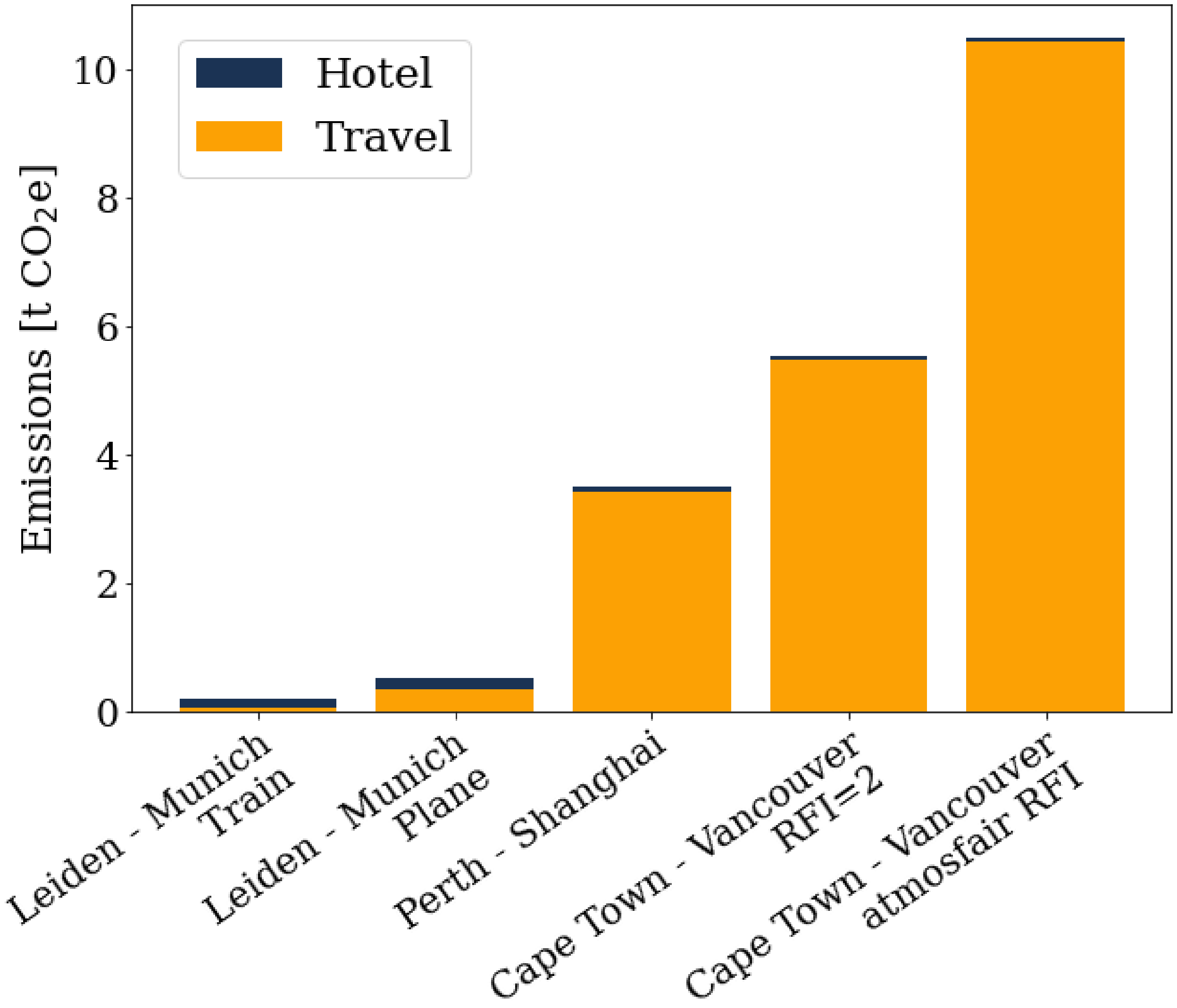

Supplementary Figure 1: Emissions for travel and hotel accommodation for three exemplary trips, each with a stay of six nights at a hotel. The contributions to the overall emissions are from travel (yellow) and accommodation (blue). To first order, flight emissions scale with distance. Train travel produces substantially lower GHG emissions than flying, and, except for longer stays in combination with train travel, the contribution by hotel accomodation is a minor fraction of total emissions. For each trip, the contribution from hotel- and travel-related emissions are, respectively: 71.6% / 28.4% (Leiden - Munich by train); 29.8% / 70.2% (Leiden - Munich by plane); 2.7% / 97.3% (Perth - Shanghai); 1.0% / 99.0% (Cape Town - Vancouver with RFI=2); 0.5 % / 99.5%(Cape Town - Vancouver with atmosfair RFI value). When comparing the impact of GHG emissions, the assumed radiative forcing index (RFI) for flights is important, as well assumptions on flight route length vs. great circles, and, potentially, the type of aircrafts. An RFI value of 1.95, as assumed throughout this study, is on the lower end of potential impact factors. Flight emission calculations through the atmosfair.de website are taking into account a higher RFI as well as typical aircraft types and real-world flight routes, which can result in substantially higher emission values.

the relative GHG emissions of accommodation versus travel we used data provided to us by atmosfair, who created and maintain emission calculation standards not only for means of travel, but also hotels[1]. A typical hotel stay can emit from below 5 $kgCO_2$ per night for a standard single room of a 1- or 2-star hotel to beyond 40 $kgCO_2$ in a 4- or 5-star hotel. The emission will vary strongly between countries with low- and high fossil-fuel use for electricity and heating or cooling, and between smaller and larger rooms. Also, these are average numbers and will depend on the setup and situation of any specific hotel.

Depending on location, 5–40 $kgCO_2$/night over a number of nights amount to emission levels that can be comparable to those of travelling to a meeting location by rail, but are typically dwarfed by flight emissions. In Supplementary Figure 1 we show the contributions of emissions for travel and accommodation for a couple of representative trips with a 6-night stay: a $\sim$800 km trip inside Europe from Leiden/Netherlands to Munich/Germany by train and plane, as well as trips from Australia to China, and South Africa to Canada.

For the intra-Europe train trip the accommodation will make up 55–70% of combined emissions — 20–30% when flying — but for the intercontinental trips it contributes only a few percent. This is, of course, due to the travel emissions being higher by factors of 10–25 in the case of intercontinental trips.

As a rule of thumb, we conclude that a hotel night has a typical GHG emission similar to $\sim$400 km of train travel, or 60–100 km short-distance flying.

Emission contribution by travel distance

In order to compute the environmental impact depending on the type of travel, we investigate how the number of short-, medium-, and long-haul travel relates to the fraction of GHG emissions associated with each category. For simplicity, we combine long-haul and ultra-long haul travel, since only a very small fraction of travel is longer than 16 hours (0.2%). The distribution is shown in Supplementary Figure 2. Almost two thirds of all trips are short distance travel, which contributes about a fifth to the GHG emissions. Long and ultra-long distance travel, which is often intercontinental, constitutes 21% of all trips, but causes more than 60% of all travel-related emissions. Medium distance trips make up the smallest fraction of all trips (13%), and the related emissions make up almost another 20%.

Lists of all astronomy conferences and schools in 2019

In our analysis we consider 362 meetings, among which were 307 conferences and 55 schools. We list all countries hosting either conferences, schools, or both in Supplementary Table 1, sorted by continent. Supplementary Tables 2 and 3 list information for each individual conference and school, respectively, for which we could obtain information about the participants' origin that enabled us to assess the travel-related GHG emissions. Conferences and schools for which we were not able to retrieve the necessary information about participants are listed in Supplementary Tables 4 and 5.

[1]Based on emission data by atmosfair gGmbH, Germany, "VDR Standard Part III", `https://www.atmosfair.de/en/standards/emissions_calculation/co2_reporting_for_companies/vdr_standard_methodology`

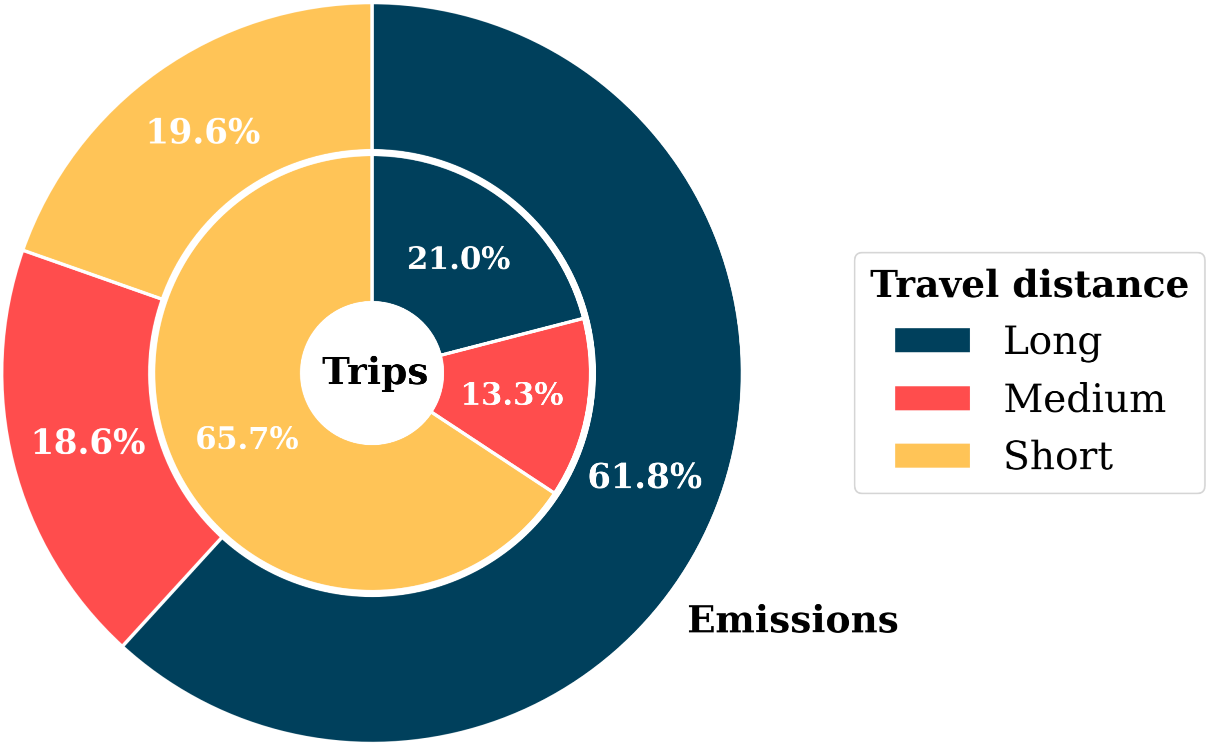

Supplementary Figure 2: Nested donut charts displaying the number of conference trips taken and divided by classification (short-, medium-, and long-haul) in the inner ring, versus the total emissions produced for each category in the outer ring. The 'long-haul' classification includes trips with ultra-long distances, which make up 0.2% of all trips and 1.3% of all emissions.

Supplementary Table 1: Distribution of conferences and schools over different continents and countries.

Continent	Number of meetings	Country	Conferences	Schools
Africa	4	Ethiopia	1	0
		Nigeria	0	1
		South Africa	2	0
Asia	61	Armenia	4	1
		Azerbaijan	1	0
		China	17	1
		India	9	8
		Iran	1	0
		Israel	2	0
		Japan	13	0
		Kazakhstan	1	0
		Malaysia	0	1
		Russia	4	2
		South Korea	1	0
		Taiwan	4	1
		Thailand	0	3
		Turkey	2	0
		Vietnam	1	0
Europe	142	Austria	1	0
		Belgium	1	0
		Bulgaria	1	0
		Czech Republic	1	0
		Denmark	1	1
		Finland	2	0
		France	9	6
		Germany	21	3
		Greece	7	1
		Hungary	3	0
		Iceland	1	0
		Ireland	1	0
		Italy	25	3
		The Netherlands	21	2
		Poland	1	3
		Portugal	3	1
		Spain	14	3
		Sweden	3	0
		Switzerland	4	2
		Ukraine	1	0
		United Kingdom	22	1
North America & Caribbean	65	Canada	8	0
		Cuba	1	0
		Mexico	2	0
		United States	63	6
Oceania	21	Australia	18	3
		New Zealand	2	0
South America	7	Argentina	2	1
		Brazil	2	1
		Chile	3	0

Supplementary Table 2: List of all 258 conferences with participant data. The number of participants given in parentheses are the amount of local participants, which travelled less than 200 km per round-trip.

Meeting name	Venue	Number of participants	Tot. travel emissions (tCO_2e)	Emission per participant (tCO_2e)	Distance trav. by train	Note
		Africa				
Cosmology on Safari	KwaZulu-Natal, South Africa	76 (12)	175.9	2.30	$<$ 300 km	
Modeling Meerkats: Comparing galaxy formation simulations to MeerKAT surveys	Kruger National Park, South Africa	22 (0)	37.61	1.71	$<$ 300 km	
IAU Symposium 356: Nuclear activity in galaxies	Addis Ababa, Ethiopia	180 (20)	301.28	1.67	$<$ 300 km	One origin not back-traceable
		Asia				
5th China-Chile Bi-lateral Astro meeting	Kunming, China	76 (10)	186.63	2.46	$<$ 700 km	
PERC International Symposium on Dust & Parent bodies (IDP 2019)	Tokyo, Japan	35 (15)	34.32	0.98	$<$ 700 km	
The Metrewavelength Sky - II: Celebrating the 90th year of Govind Swarup and the 1st year of the upgraded GMRT	Pune, India	187 (69)	220.03	1.18	$<$ 500 km	
Cosmic Lab	Narashino, Japan	17 (8)	9.16	0.54	$<$ 700 km	
Search for life, from Early Earth to Exoplanets	Quy Nhon, Vietnam	49 (2)	131.54	2.68	$<$ 300 km	
Extremely big Eyes on the early Universe	Kashiwa, Japan	78 (22)	87.52	1.12	$<$ 500 km	
The 1st Donglu Astrophysics Forum The era of large-scale sky surveys: Near field cosmology and time domain science	Kunming, China	121 (36)	55.30	0.46	$<$ 1000 km	
VHE Phenomena Around Supermassive Black Holes	Yerevan, Armenia	47 (3)	65.63	1.40	$<$ 300 km	
The 9th International Workshop on Occultation and Eclipse (IWOE9)	Canakkale, Turkey	40 (8)	16.29	0.41	$<$ 1000 km	
The correlated Universe	Seogwipo, South Korea	38 (0)	42.65	1.12	$<$ 100 km	
Tal Alexander workshop: Dynamics and accretion near massive black holes	Tel Aviv, Israel	63 (49)	30.18	0.48	$<$ 100 km	
Recent progress in relativistic astrophysics	Shanghai, China	88 (4)	112.35	1.28	$<$ 1000 km	
International Pulsar Timing Array Meeting - 2019	Pune, India	94 (13)	220.88	2.35	$<$ 700 km	
Exploring the Energetic Universe 2019	Astana, Kasakhstan	16 (4)	20.95	1.31	$<$ 300 km	
Future Science with Multi-Wavelength Data: North Ecliptic Pole Meeting 2019	Hsinchu, Taiwan	45 (20)	32.37	0.72	$<$ 300 km	
IAU Symposium 353: Galactic Dynamics in the Era of Large Surveys	Shanghai, China	81 (9)	219.91	2.71	$<$ 1000 km	Participants estimated from programme
Cosmic Dust	Narashino, Japan	57 (14)	76.51	1.34	$<$ 700 km	
From AGN to starburst	Guiyang, China	107 (3)	155.07	1.45	$<$ 700 km	
Gravity meets Plasma Workshop	Kunming, China	44 (0)	59.88	1.36	$<$ 1000 km	
Dynamics of asteroids, TNOS & natural satellites with Gaia data	Antalya, Turkey	29 (12)	25.58	0.88	$<$ 300 km	
Active galaxies and quasars	Byurakan, Armenia	22 (15)	3.52	0.16	$<$ 300 km	
KIAA Forum on Gas in Galaxies "Multiple-phase Interstellar medium: Probing the Activities and Power Engines from Local to Distant Universe"	Beijing, China	100 (41)	111.44	1.11	$<$ 1000 km	
IAU South West & Asian regional workshop	Byurakan, Armenia	37 (16)	11.90	0.32	$<$ 300 km	
Compact white dwarf binaries	Yerevan, Armenia	64 (3)	135.63	2.12	$<$ 300 km	
Challenges and innovations in computational astrophysics	Saint Petersburg, Russia	102 (3)	84.40	0.83	$<$ 700 km	
Physics of Stars and Planets: Atmospheres, Activity, Magnetic fields	Shamakhy, Azerbaijan	111 (42)	43.35	0.39	$<$ 300 km	
Mapping Central regions of AGN	Shanghai, China	122 (4)	233.36	1.91	$<$ 1000 km	
Multi-messenger astrophysics in gravitational wave era	Kyoto, Japan	165 (28)	259.62	1.57	$<$ 700 km	
Diversity of the local Universe	Nizhnij Arkhyz, Russia	82 (23)	78.26	0.95	$<$ 500 km	
The UX Ori type stars and related topics	Saint Petersburg, Russia	59 (13)	47.79	0.81	$<$ 300 km	
Second Workshop on Numerical Modeling in MHD and Plasma Physics: Methods, Tools, and Outcomes	Moscow, Russia	29 (12)	17.82	0.61	$<$ 500 km	
The Milky Way 2019: LAMOST and Other Leading Surveys	Yichang, China	110 (3)	85.72	0.78	$<$ 1000 km	
New Horizons in Galactic Center Astronomy and Beyond	Yokohama, Japan	136 (37)	230.37	1.69	$<$ 700 km	
Cosmic Evolution of Quasars: from the First Light to Local Relics	Beijing, China	112 (28)	144.23	1.29	$<$ 1000 km	
Gamma-ray Bursts in the Gravitational Wave Era 2019	Yokohama, Japan	161 (51)	272.47	1.69	$<$ 700 km	
TMT Science Forum	Xiamen Island, China	113 (9)	160.35	1.42	$<$ 1000 km	Origin for some participants not clear
4th COSPAR Symposium: Small Satellites for Sustainable Science and Development	Herzliya, Israel	258 (63)	434.16	1.68	$<$ 300 km	
1st Shanghai assembly on cosmology & galaxy	Shanghai, China	100 (26)	111.15	1.11	$<$ 1000 km	
Science with the Submillimeter Array: Present and Future	Taipei, Taiwan	108 (51)	118.65	1.10	$<$ 500 km	
JCMt Users	Taipei, Taiwan	87 (40)	74.72	0.86	$<$ 300 km	
Astronomy for equity, diversity and inclusion	Tokyo, Japan	121 (16)	289.1	2.39	$<$ 700 km	
International Conference on Modeling, Machine Learning and Astronomy	Bangalore, India	150 (20)	71.03	0.47	$<$ 700 km	Cities for origin of participants estimated
Planet formation workshop	Tokyo, Japan	88 (36)	71.02	0.81	$<$ 700 km	
International conference on Gravitation & Cosmology	Punjab, India	196 (1)	141.75	0.72	$<$ 500 km	
Young Astronomers' Meet 2019 at Kodaikanal Solar Observatory	Kodaikanal, India	57 (0)	21.26	0.37	$<$ 700 km	
Modern Engineering Trends in Astronomy - 2019(META-2019)	Bangalore, India	193 (107)	25.14	0.13	$<$ 700 km	
Multi-wavelength Astronomy: Three years of AstroSat observations	Bangalore, India	84 (50)	12.96	0.15	$<$ 300 km	
International Symposium on Extra - Solar Planets	Bangalore, India	20 (5)	19.98	1.00	$<$ 300 km	
2019 ASROC Annual Meeting	Taichung, Taiwan	176 (84)	11.23	0.06	$<$ 500 km	

Table 2 continued.

Meeting name	Venue	Number of participants	Tot. travel emissions (tCO_2e)	Emission per participant (tCO_2e)	Distance trav. by train	Note
		Europe				
The Supernova-Supernova Remnant Connection	London, UK	16 (4)	3.64	0.23	< 300 km	Only origin countries were known
Spectroscopy with SOFIA: new results & future opportunities	Kreuth, Germany	59 (2)	39.14	0.66	< 500 km	
Monsters of the Universe: The Most Extreme Star Factories	Leiden, Netherlands	36 (0)	28.31	0.79	< 500 km	
Nucleosynthesis for a Life: Symposium in honour of Prof. Roland Diehl	Garching, Germany	60 (50)	3.69	0.06	< 500 km	
12th INTEGRAL conference	Geneva, Switzerland	166 (11)	115.14	0.69	< 500 km	
Formation of Stars and Massive clusters in Dwarf Galaxies over Cosmic Time	Leiden, Netherlands	45 (5)	44.54	0.99	< 500 km	Only origin contintents were known
Large survey with small telescopes	Bamberg, Germany	79 (13)	46.18	0.58	< 500 km	
20 years of FORS	Garching, Germany	12 (2)	9.12	0.76	< 500 km	
Extreme Precision in Radial Velocity IV	Grindelwald, Switzerland	121 (0)	196.05	1.62	< 300 km	
Galaxy Evolution in the Cosmic Web	Leiden, Netherlands	27 (3)	28.38	1.05	< 500 km	
The New Era of Multi-Messenger Astrophysics	Groningen, Netherlands	152 (28)	113.44	0.75	< 500 km	
Turbulence & magnetic fields	Helsinki, Finland	60 (8)	49.93	0.83	< 300 km	
SKA General Science Meeting	Macclesfield, UK	270 (64)	323.51	1.20	< 300 km	
ESLAB symposium: the Gaia universe	Noordwijk, The Netherlands	142 (21)	67.15	0.47	< 300 km	
Laboratory Astrophysics	Cambridge, UK	172 (17)	163.95	0.95	< 300 km	
Delving into the neutron star crust (ICONS)	Amsterdam, Netherlands	38 (9)	47.29	1.24	< 500 km	
PHAROS: multimessenger physics & neutron stars	Barcelona, Spain	176 (12)	123.52	0.70	< 500 km	
Metals in Galaxies, Near and Far: Looking Ahead	Leiden, Netherlands	47 (3)	51.67	1.10	custom	flights from UK, 500km rule for other countries
Preparing for 4MOST	Garching, Germany	114 (27)	60.13	0.53	< 500 km	
1st CTA Symposium	Bologna, Italy	275 (44)	249.32	0.91	< 500 km	
ExoComets: Understanding the Composition of Planetary Building Blocks	Leiden, Netherlands	56 (10)	52.85	0.94	< 700 km	
Herschel 10 years after launch	Madrid, Spain	69 (28)	34.95	0.51	< 500 km	
Multi-messenger astro with SKA	Aveiro, Portugal	65 (20)	53.80	0.83	< 500 km	
Meeting of Italian Astronomical Society	Rome, Italy	145 (58)	14.40	0.10	< 500 km	
Exploring the Infrared Universe	Heraklion, Greece	164 (4)	271.48	1.66	< 300 km	
Multi-spin galaxies	Asiago, Italy	32 (8)	30.41	0.95	< 300 km	
Workshop on Polarization in Protoplanetary Disks and Jets	Sant Cugat, Spain	58 (7)	82.27	1.42	< 500 km	
Star clusters: from the MilkyWay to the Early Universe	Bologna, Italy	204 (18)	308.42	1.51	< 500 km	
From Interstellar medium to large scale structures	Stockholm, Sweden	73 (10)	71.29	0.98	< 500 km	
Supernova remnants	Chania, Greece	193 (4)	401.31	2.08	< 300 km	
What matter(s) between galaxies: unraveling the knots in the Cosmic Web	Abbazia di Spineto, Italy	73 (0)	121.05	1.66	< 500 km	
Linking the Milky Way and Nearby Galaxies: The ISM and Star Formation from Cold Cores to Kpc Scales	Helsinki, Finland	99 (6)	165.1	1.67	< 300 km	
Planetary Dynamics 2019	Heidelberg, Germany	67 (19)	62.01	0.93	< 500 km	
Partially ionised plasmas in astrophysics	Palma de Mallorca, Spain	53 (8)	57.00	1.08	< 300 km	
IAU Symposium 352: Uncovering early galaxy evolution in the ALMA and JWST era	Viana do Castelo, Portugal	172 (6)	312.88	1.82	< 300 km	Only origin countries were known
Joint conference of sub-regional EU astro committee	Sofia, Bulgaria	56 (12)	4.38	0.08	< 500 km	
Zooming in on Star Formation	Nafplio, Greece	116 (3)	174.47	1.50	< 300 km	
New perspectives on galactic magnetism	Newcastle upon Tyne, UK	61 (6)	72.37	1.19	< 300 km	
Astrophysics of hot plasma in extended X-ray sources	Madrid, Spain	78 (6)	110.34	1.41	< 500 km	
5th workshop of SCAR AAA, Astronomy and Astrophysics from Antarctica	Courmayeur, Italy	38 (0)	73.93	1.95	< 1000 km	
The cosmic web	Edinburgh, UK	80 (14)	78.95	0.99	< 500 km	
From Stars to Planets II - Connecting our understanding of star and planet formation	Gothenburg, Sweden	161 (14)	185.98	1.16	< 300 km	
Starry	Leeds, UK	64 (7)	65.81	1.03	< 300 km	
9th meeting on hot subdwarfs	Hendaye, France	60 (0)	77.38	1.29	< 500 km	
Astrochemistry: from nanometers to megaparsecs	Gothenburg, Sweden	79 (19)	65.02	0.82	< 500 km	
The new era of multi-messenger astrophysics	Groningen, Netherlands	149 (28)	110.01	0.74	< 300 km	
Ringberg Conference on Star-Planet connection	Kreuth, Germany	49 (0)	30.74	0.63	< 700 km	
RAS National Astronomy Meeting 2019	Lancaster, UK	526 (121)	78.23	0.15	< 300 km	
Alpine Cosmology Workshop 2019 (ACW19)	La Berarde, France	20 (1)	6.54	0.33	< 500 km	
From winds to jets: the role of outflows in compacts binaries	Amsterdam, Netherlands	55 (15)	45.10	0.82	< 500 km	
The Beginnings and Ends of Double White Dwarfs	Copenhagen, Denmark	72 (21)	95.43	1.33	< 500 km	
14th International Conference on Numerical Modeling of Space Plasma Flows (ASTRONUM-2019)	Paris, France	82 (7)	136.14	1.66	< 300 km	
14th hellenic astronomical conference	Volos, Greece	139 (2)	56.57	0.41	< 300 km	
Tracing Cosmic Evolution with Clusters of Galaxies IV	Sesto, Italy	255 (1)	359.21	1.41	< 300 km	
Turbulence, Structure Formation and Accretion in Protoplanetary Disks: Theory, Observation and Experiment	Kreuth, Germany	60 (5)	61.83	1.03	< 500 km	

Table 2 continued.

Meeting name	Venue	Number of participants	Tot. travel emissions (tCO_2e)	Emission per participant (tCO_2e)	Distance trav. by train	Note
IAU Symposium 355: The Realm of the Low Surface Brightness Universe	La Laguna, Spain	120 (15)	201.05	1.68	$<$ 100 km	Only origin countries were known
High Energy phenomena in relativist outflows	Barcelona, Spain	97 (13)	106.30	1.10	$<$ 500 km	
Astronomy in Ukraine: from archaeoastronomy to high-energy astrophysics.	Kyiv, Ukraine	71 (45)	11.38	0.16	$<$ 300 km	
XCalibur 2019 – High resolution X-ray spectroscopy as a probe of strong gravity and dark matter: From Hitomi to XRISM and beyond to Athena	Southampton, UK	74 (10)	75.32	1.02	$<$ 300 km	
A centenary of astrohpysical jets	Cheshire, UK	119 (17)	128.8	1.08	$<$ 300 km	
Understanding cosmological observations	Benasque, Spain	65 (0)	73.32	1.13	$<$ 500 km	
Nine Billion Years of Neutral Gas Evolution	Garching, Germany	84 (15)	151.08	1.80	$<$ 500 km	
Small galaxies, cosmis questions	Durham, UK	131 (20)	188.91	1.44	$<$ 300 km	
Quasars in crisis	Edinburgh, UK	69 (10)	86.40	1.25	$<$ 300 km	
Improving Image Fidelity on Astronomical Data: Radio Interferometer and Single-Dish Data Combination	Leiden, Netherlands	44 (7)	59.55	1.35	$<$ 500 km	
Extreme solar systems IV	Reykjavik, Iceland	583 (2)	928.25	1.59	$<$ 100 km	
Stars and their Variability, Observed from Space	Vienna, Austria	256 (30)	249.95	0.98	$<$ 500 km	Based on country of nationality
Stars on the Run II	Potsdam, Germany	70 (16)	43.03	0.61	$<$ 500 km	
Astro Hack Week 2019	Cambridge, UK	34 (4)	47.65	1.40	$<$ 300 km	Participant list $\sim 40\%$ incomplete
49th Young European Radio Astronomers Conference	Dublin, Ireland	34 (7)	13.57	0.40	$<$ 300 km	
Matera Oscura	Matera, Italy	139 (2)	145.76	1.05	$<$ 500 km	
Physics & chemisty of interstellar medium	Avignon, France	123 (0)	135.71	1.10	$<$ 500 km	
Views on interstellar medium in galaxies with ALMA	Bologna, Italy	116 (23)	105.43	0.91	$<$ 500 km	
Variable Galactic Gamma-Ray Sources (VGGRS) V	Barcelona, Spain	45 (8)	51.01	1.13	$<$ 300 km	
Astrophysics with GW detections	Warsaw, Poland	58 (22)	40.89	0.70	$<$ 500 km	
Universe of Binaries, Binaries in the Universe	Telc, Czech Republic	88 (7)	91.54	1.04	$<$ 300 km	
X-ray astronomy 2019	Bologna, Italy	315 (35)	324.60	1.03	$<$ 500 km	
A synoptic view of the magellanic clouds	Garching, Germany	104 (15)	165.45	1.59	$<$ 500 km	
Stars as probes of first-star nucleosynthesis	Geneva, Switzerland	78 (18)	113.94	1.46	$<$ 700 km	
Extremely big eyes on the early universe	Rome, Italy	96 (21)	70.32	0.73	$<$ 500 km	
Nuclear Physics in Astrophysics IX	Frankfurt, Germany	139 (51)	92.44	0.67	$<$ 500 km	Abstract book used for affiliations
IAU Astronomy Education Conference: Bridging Research & Practice	Garching, Germany	114 (4)	150.45	1.32	$<$ 500 km	Only origin countries were known
From Gas to stars	York, UK	82 (17)	81.30	1.00	$<$ 500 km	
The 3C extragalactic radio sky	Torino, Italy	98 (17)	106.36	1.09	$<$ 500 km	
The extragalactic explosive universe	Garching, Germany	102 (14)	88.14	0.86	$<$ 300 km	
Meeting of Astronomische Gesellschaft 2019	Stuttgart, Germany	219 (64)	48.25	0.22	$<$ 700 km	
Cosmology: the end of the beginning. Future prospects in cosmology, large scale structure and galaxy formation	Cambridge, UK	153 (56)	141.18	0.92	$<$ 300 km	
Statistics to the Stars and Back: Convincing Inference Across the Interdisciplinary Gap	Leiden, Netherlands	32 (25)	0.81	0.03	custom	Data available on how may trains and planes were taken
Rotation periods of cool stars	Potsdam, Germany	54 (14)	45.65	0.85	$<$ 500 km	
Crete III - Through dark lanes to new stars: Celebrating the career of Prof. Charles Lada	Crete, Greece	93 (3)	183.23	1.97	no train	
Bonn Gravity 2019	Bonn, Germany	74 (26)	61.93	0.84	$<$ 500 km	
The time machine factory	Torino, Italy	82 (29)	39.78	0.49	$<$ 500 km	
YAGN19 - Young Astronomers on Galactic Nuclei	La Laguna, Spain	29 (5)	32.84	1.13	$<$ 300 km	
The legacy of the gaia-eso survey	Firenze, Italy	89 (18)	75.00	0.84	$<$ 500 km	
38th International Meteor Conference	Bollmannsruh, Germany	99 (11)	39.07	0.39	$<$ 500 km	
ADASS	Groningen, Netherlands	350 (67)	438.54	1.25	$<$ 500 km	
Untangle the skein with Scarlet (LSST)	Rome, Italy	37 (6)	9.17	0.25	$<$ 300 km	
Cosmic Ray anisotropy workshop	Rome, Italy	58 (16)	53.11	0.92	$<$ 500 km	
RAS Specialist Discussion Meeting: Common envelope evolution and post-common-envelope systems	London, UK	40 (13)	6.41	0.16	$<$ 300 km	Particpants based on statement of organisers, assuming most participants are from UK
SKA-VLBI Key Science workshop	Cheshire, UK	65 (19)	86.59	1.33	$<$ 500 km	
ALMA2019: science results and cross-facility synergies	Cagliari, Italy	235 (3)	433.38	1.84	no train	
Historical Supernovae, Novae and Other Transient Events	Leiden, Netherlands	30 (5)	31.26	1.04	$<$ 700 km	
The Future of X-ray Timing	Amsterdam, Netherlands	123 (27)	122.49	1.00	$<$ 500 km	
First Galaxies, First Structures	Paris, France	60 (15)	57.17	0.95	$<$ 500 km	
EMU international meeting	Catania, Italy	27 (8)	40.26	1.49	$<$ 500 km	
Revolutionary Spectroscopy of Today as a Springboard to Webb	Leiden, Netherlands	54 (4)	86.66	1.60	$<$ 700 km	
Voyage 2050 workshop - Shaping the European Space Agency's space science plan for 2035-2050	Madrid, Spain	262 (1)	159.43	0.61	$<$ 300 km	Only origin continents were known
Scintillometry 2019	Bonn, Germany	63 (14)	90.16	1.43	$<$ 500 km	
First Spanish Meeting on Galaxy Clusters	Madrid, Spain	41 (15)	16.16	0.39	$<$ 500 km	
Lithium in the Universe: to Be or not to Be?	Rome, Italy	84 (6)	104.84	1.25	$<$ 500 km	
The art of measuring galaxy physical properties	Milano, Italy	133 (8)	182.68	1.37	$<$ 500 km	
PHYSICS and ASTROPHYSICS of COSMIC RAYS	Saint Michel l'Observatoire, France	40 (5)	15.02	0.38	$<$ 500 km	
WorkPlaNS II: Workshop for Planetary Nebula observations	Leiden, Netherlands	50 (3)	72.02	1.44	$<$ 500 km	
The Origins of Black Hole Mergers and Gravitational	Leiden, Netherlands	35 (9)	51.40	1.47	$<$ 500 km	

Table 2 continued. [†]Origin cities used for US states are state capitols, except for the following states, for which we took the cities given in parentheses instead: Alabama (Tuscaloosa), Arizona (Tucson, Flagstaff), California (Los Angeles, San Diego, San Francisco), Connecticut (New Haven), Illinois (Chicago), Maryland (Baltimore), Michigan (Lansing, Ann Arbor), Minnesota (Minneapolis), Missouri (Saint Louis), New Mexico (Albuquerque, Socorro), New York (Albany, Ithaca, New York City, Rochester), Pennsylvania (Philadelphia, State College), South Carolina (Clemson, Columbia), Texas (Austin, Houston, Dallas), Virginia (Charlottesville, Lynchburg), Washington (Bellingham, Pullman, Seattle), and West Virginia (Morgantown). For states with more than two listed cities we divided the number of participants equally.

Meeting name	Venue	Number of participants	Tot. travel emissions (tCO_2e)	Emission per participant (tCO_2e)	Distance trav. by train	Note
10th OPTICON Gaia Science Alerts Workshop	Catania, Italy	54 (5)	37.67	0.70	< 500 km	
5ème atelier PLATO/France: Synergies PLATO/ARIEL	Bordeaux, France	44 (5)	2.22	0.05	< 700 km	
AO4ASTRO	Marseille, France	62 (23)	28.81	0.46	< 500 km	
TRAPPIST-1: Towards the Comparative Study of Temperate Terrestrial Worlds	Liège, Belgium	90 (23)	82.95	0.92	< 300 km	
Interstellar Exploration	Noordwijk, Netherlands	30 (16)	0.43	0.01	custom	Data included how may trains and planes were taken
Exoclimes V	Oxford, UK	139 (24)	192.2	1.38	< 300 km	
European Planetary Science Congress - AAS Division Planetary Science Joint Meeting	Geneva, Switzerland	1730 (52)	2341.71	1.35	< 500 km	Only origin countries were known
Wavefront sensing in the VLT/ELT era	Firenze, Italy	81 (24)	33.42	0.41	< 500 km	
MW-Gaia WG3 Workshop: Exoplanets in the era of Gaia	Porto, Portugal	36 (10)	16.32	0.45	< 500 km	
EAS Meeting 2019	Lyon, France	1462 (0)	1034.80	0.71	< 500 km	
		North & Central America				
233rd AAS meeting	Seattle, WA, USA	3396 (118)	3461.97	1.02	< 300 km	Only origin country and US states[†] were known
Testing Gravity 2019	Vancouver, Canada	117 (26)	159.70	1.36	< 300 km	
23rd International Microlensing Conference	Pasadena, CA, USA	82 (16)	133.90	1.63	< 300 km	Data for 2019 conference not available, used 2017 participant list
Extremely big eyes on the early universe	Los Angeles, CA, USA	70 (17)	54.74	0.78	< 300 km	
Massively multiplexed spectroscopy with MSE	Tucson, AZ, USA	93 (11)	122.9	1.32	< 300 km	
Into the Starlight: The End of the Cosmic Dark Ages	Aspen, CO, USA	61 (0)	98.08	1.61	< 300 km	
Dusting the Universe	Tucson, AZ, USA	82 (7)	125.24	1.53	< 300 km	Origin for one participant not retrievable
Kepler & K2 Science Conference V	Glendale, CA, USA	283 (67)	331.27	1.17	< 300 km	
HEAD 17th Divisional Meeting	Monterey, CA, USA	362 (25)	444.7	1.23	< 300 km	
CHARA 2019	Flagstaff, AZ, USA	47 (15)	36.20	0.77	< 500 km	
r-process sources in the universe	Tempe, AZ, USA	37 (6)	38.82	1.05	no train	
New quests in stellar astrophysics IV	Puerto Vallarta, Mexico	109 (0)	141.48	1.30	< 300 km	
The Landscape of Space Astrophysics in the 2020s and Beyond	Potomac, MD, USA	84 (47)	30.84	0.37	< 100 km	Participants estimated from programme
Better Stars, Better Planets: Exploiting the Stellar-Exoplanetary Synergy	Santa Barbara, CA, USA	69 (6)	98.78	1.43	< 300 km	
The Deaths and Afterlives of Stars	Baltimore, MD, USA	143 (28)	161.64	1.13	< 300 km	
The Future of Canadian Radio Astronomy	Penticton, Canada	45 (12)	19.76	0.44	< 300 km	
Stars 2019	Havana, Cuba	53 (14)	81.75	1.54	< 300 km	
New Horizons in planetary systems	Victoria, Canada	144 (21)	192.95	1.34	< 500 km	
The New Era of Gravitational-Wave Physics and Astrophysics	Santa Barbara, CA, USA	69 (6)	100.02	1.45	< 300 km	
Canada-France-Hawaii Telescope 12th Users' Meeting	Montreal, Canada	130 (38)	142.8	1.10	< 300 km	
F.O.E. Fifty-One Erg Supernova Conference	Raleigh, NC, USA	116 (16)	196.38	1.69	< 300 km	
Memorial Symposium to honor Riccardo Giacconi	Washington DC, USA	106 (44)	67.80	0.64	custom	data with means of transportation were provided
234th AAS meeting	St. Louis, MO, USA	753 (71)	511.97	0.68	< 300 km	Only origin country and US states[†] were known
2019 Canadian Astronomical Society Annual Meeting	Montreal, Canada	302 (85)	184.19	0.61	< 300 km	
Exploring the Galaxy and the Local Group with WFIRST	Pasadena, CA, USA	79 (45)	38.69	0.49	< 300 km	
Gordon Conference on Origins of Solar Systems	South Hadley, MA, USA	121 (4)	136.50	1.13	< 300 km	
Astroinformatics 2019	Pasadena, CA, USA	59 (32)	61.60	1.04	< 300 km	
SDSS-IV/V Collaboration meeting	Ensenada, Mexico	142 (37)	164.02	1.16	< 300 km	
Radio/Millimeter Astrophysical Frontiers next decade	Charlottesville, VA, USA	115 (41)	61.41	0.53	< 300 km	
Kavli Summer Program In Astrophysics 2019: Machine Learning in the era of large astronomical surveys	Santa Barbara, CA, USA	35 (0)	60.84	1.74	< 300 km	
Space climate 7	Canton Orford, Canada	76 (0)	119.58	1.57	< 500 km	
TASC5/KASC12 workshop	Cambridge, MA, USA	118 (13)	204.24	1.73	< 300 km	
TESS Science Conference I	Cambridge, MA, USA	282 (92)	316.03	1.12	< 300 km	
Long Wavelength Array Users Meeting	Albuquerque, NM, USA	42 (14)	15.01	0.36	< 300 km	
Multiscale Phenomena in Plasma Astrophysics	Santa Barbara, CA, USA	75 (1)	134.14	1.79	< 300 km	Participants estimated from conference picture
LSST 2019 Project and Community Workshop	Tucson, AZ, USA	300 (106)	241.34	0.80	no train	
VISTA Variables in Via Lactea (VVV) 10th Science meeting.	Hilo, HI, USA	25 (9)	53.28	2.13	< 100 km	
Hot-wiring the transient universe VI	Evanston, IL, USA	93 (19)	99.82	1.07	< 300 km	
Non-Standard Cosmology Probes	Aspen, CO, USA	40 (0)	76.32	1.91	no train	
Connecting Micro and Macro Scales: Acceleration, Reconnection, and Dissipation in Astrophysical Plasmas	Santa Barbara, CA, USA	76 (5)	129.00	1.70	< 300 km	
The Cosmic Baryon Cycle: Impact on Galaxy Evolution	Carlsbad, CA, USA	103 (12)	137.16	1.33	< 300 km	
Midwest workshop on Supernovae and transients	Columbus, OH, USA	67 (24)	17.81	0.27	< 300 km	

Table 2 continued.

Meeting name	Venue	Number of participants	Tot. travel emissions (tCO_2e)	Emission per participant (tCO_2e)	Distance trav. by train	Note
Managing Follow-up Observations in the Era of ZTF and LSST	Pasadena, CA, USA	51 (11)	83.01	1.63	< 300 km	
Cosmic controversies	Chicago, IL, USA	281 (98)	204.87	0.73	< 300 km	
Warm Ionized Medium in Galaxies Workshop	Green Bank, WV, USA	45 (6)	29.43	0.65	< 100 km	
The RR Lyrae and Cepheid Conference 2019: Frontiers of Classical Pulsators - Theory and Observations	Cloudcroft, NM, USA	75 (0)	165.59	2.21	no train	
IAU Symposium 357: White Dwarfs as probes of fundamental physics and tracers of planetary, stellar and galactic evolution	Hilo, HI, USA	93 (9)	250.40	2.69	no train	
Rocky Exoplanets in the era of JWST	Greenbelt, MD, USA	125 (70)	58.66	0.47	< 300 km	
The New Faces of Black Holes, 2019 JSI Workshop	Annapolis, MD, USA	70 (21)	45.05	0.64	< 300 km	Participants taken from picture, their origin from contributions
Subaru Telescope 20th Anniversary - Optical & Infrared Astronomy for the Next Decade	Waikoloa, HI, USA	240 (35)	453.88	1.89	< 100 km	
Universality: Turbulence across vast scales	New York City, NY, USA	67 (30)	43.99	0.66	< 300 km	
Twenty Years of Science with Chandra	Boston, MA, USA	285 (154)	172.85	0.61	< 300 km	
Science at Low Frequencies VI	Tempe, AZ, USA	132 (17)	260.86	1.98	< 300 km	
Keck Wide-Field Imager Science Workshop	Pasadena, CA, USA	41 (13)	61.41	1.50	< 300 km	
TESS Data Workshop	Baltimore, MD, USA	80 (10)	72.87	0.91	< 100 km	No data on origin cities
Telluric Line Hack Week	New York City, NY, USA	44 (3)	43.62	0.99	< 300 km	
Chesapeake Bay Area Exoplanet Meeting	Baltimore, MD, USA	60 (55)	0.66	0.01	< 300 km	
Planet-Star Connections in the Era of TESS and Gaia	Santa Barbara, CA, USA	70 (8)	114.14	1.63	< 100 km	No data on origin cities
2019 Mid-American Regional Astrophysics Conference (MARAC)	Atchison, USA	50 (17)	6.30	0.13	< 300 km	
		Oceania				
12th PHISCC workshop	Perth, Australia	80 (24)	167.17	2.09	< 300 km	
Linking galaxies from the epoch of initial star formation to today	Sydney, Australia	162 (57)	437.36	2.70	< 500 km	Uncertainty for city origin within Australia
The life and death of starforming regions	Perth, Australia	91 (16)	306.4	3.37	< 300 km	
LSST@Asia	Sydney, Australia	102 (9)	257.60	2.53	< 300 km	
Barefoot reionization	Fitzroy Island, Australia	85 (0)	278.66	3.28	< 300 km	
Great Barriers in Planet Formation	Palm Cove, Australia	115 (0)	461.27	4.01	no train	
Astronomical Data Archives	Sydney, Australia	42 (9)	86.80	2.07	< 300 km	
New Zealand Starlight Conference	Lake Tekapo, New Zealand	132 (11)	142.37	1.08	< 300 km	
Exploiting Large Radio and mm Surveys to understand the ISM	Kingscliff, Australia	22 (0)	14.12	0.64	< 300 km	
New Zealand Square Kilometre Array Forum	Auckland, New Zealand	48 (24)	28.11	0.59	no train	
ACAMAR5 - Healesville	Melbourne, Australia	82 (18)	139.89	1.71	no train	
Dynamical Models of Observed Galaxies	Sydney, Australia	25 (15)	11.81	0.47	no train	
ASA ASM	Brisbane, Australia	233 (25)	147.41	0.63	no train	
Astronomical Data Archives	Sydney, Australia	42 (12)	88.48	2.11	no train	
Stars in Melbourne Workshop	Melbourne, Australia	37 (19)	12.67	0.34	no train	
MAVIS Workshop	Sydney, Australia	19 (12)	3.22	0.17	no train	
ASTRO 3D annual retreat	Ballarat, Australia	95 (0)	54.26	0.57	< 300 km	
ASTRO 3D science meeting	Sydney, Australia	86 (23)	56.34	0.66	no train	
		South America				
The La Silla Observatory - inauguration to future	La Serena, Chile	73 (7)	168.06	2.30	< 500 km	
9th Friends of Friends Meeting	Cordoba, Argentina	133 (84)	84.80	0.64	< 500 km	
Workshop de Infraestructura Astronómica argentina	Cordoba, Argentina	83 (46)	16.14	0.19	< 300 km	
Precision spectroscopy workshop	Sao Paulo, Brazil	43 (27)	14.54	0.34	< 500 km	
IAU meeting: Regional Latin-American	Antofagasta, Chile	330 (29)	247.57	0.75	< 500 km	

Supplementary Table 3: List of all 42 schools with participant data. The number of participants given in parentheses are the amount of local participants, which travelled less than 200 km per round-trip.

Meeting name	Venue	Number of participants	Tot. travel emissions (tCO_2e)	Emission per participant (tCO_2e)	Distance trav. by train	Note
**Africa**						
West African International Summer School for Young Astronomers	Abuja, Nigeria	70 (11)	32.65	0.47	< 300 km	Estimated origin cities for students from Nigeria
**Asia**						
Cosmogly – The Next Decade	Bangalore, India	43 (7)	48.03	1.12	< 700 km	
The 1st Thai-CTA workshop on Astroparticle Physics	Chiang Mai, Thailand	74 (8)	46.00	0.62	< 300 km	
Astrowin19 - Winter School on Astronomy	Hyderabad, India	64 (11)	33.67	0.53	< 500 km	
A COSPAR capacity building workshop	Punjab, India	35 (1)	22.49	0.64	< 300 km	
NARIT-EACOA Summer Workshop on Astrostatistics & Astroinformatics	Chiang Mei, Thailand	54 (7)	36.77	0.68	< 300 km	
IFAS5 - Astronomy summer school - Spectroscopy & Spectrographs	Pune, India	31 (14)	13.79	0.44	< 300 km	
Regional summer school on space sciences	Byurakan, Armenia	23 (15)	5.43	0.24	< 300 km	
1st Malaysian VLBI Workshop	Kuala Lumpur, Malaysia	54 (29)	28.37	0.53	< 300 km	
APCTP School/Workshop on Gravitational-Wave Cosmology	Taipei, Taiwan	100 (35)	81.84	0.82	< 300 km	
Solar Physics Summer School at Raman Science Center, Leh	Leh, India	50 (0)	56.78	1.14	< 300 km	
42nd International school for Young Astronomers	Kunming, China	64 (23)	44.41	0.69	< 1000 km	
8th IIST Astronomy and Astrophysics School - 2019	Thiruvananthapuram, India	47 (1)	17.08	0.36	< 500 km	
**Europe**						
The Milky Way in the Gaia Era	Saas Fee, Switzerland	38 (0)	48.68	1.28	< 500 km	
Gravitational wave open data workshop	Paris, France	54 (18)	30.50	0.56	< 500 km	
Multiple approaches to plasma physics	Les Houches, France	70 (1)	57.28	0.82	< 700 km	
Advanced school on exoplanetary science	Salerno, Italy	86 (5)	72.80	0.85	< 500 km	
Cosmic explosions 2019	Cargese, France	54 (0)	51.85	0.96	< 100 km	Only country of origin known
SPACE ASTROMETRY FOR ASTROPHYSICS	L'Aquila, Italy	55 (1)	70.70	1.29	< 500 km	
Cracow school of Theoretical physics	Zakopane, Poland	69 (44)	26.43	0.38	< 500 km	
Torun Astrophysics, Spectroscopy and Quantum Chemistry school	Torun, Poland	60 (19)	25.20	0.42	< 500 km	
Artificial Intelligence in Astronomy	Garching, Germany	122 (28)	101.80	0.83	< 500 km	
Summer school on protoplanetary disks	Copenhagen, Denmark	53 (15)	42.62	0.80	< 500 km	
IRAM 30m summer school	Granada, Spain	43 (4)	39.43	0.92	< 500 km	
Heidelberg summer school: Instrumentation for Ground-based Optical & Infrared Astronomy	Heidelberg, Germany	51 (10)	41.13	0.81	< 500 km	
Torun summer school: Polarimetry as diagnostic tool in astronomy	Torun, Poland	34 (9)	13.04	0.38	< 500 km	
School of Statistics for Astrophysics 2019: Variability and Time Series Analysis	Autrans, France	20 (5)	2.71	0.14	< 500 km	
1st IAA-CSIC Severo Ochoa School on Statistics, Data Mining and Machine Learning	Granada, Spain	45 (20)	25.89	0.58	< 500 km	
NYRIA workshop 2019	Lisbon, Portugal	19 (3)	15.14	0.80	< 300 km	
CNRS thematic school of astroparticle physics	Manosque, France	40 (5)	15.02	0.38	< 500 km	
Astroparticle School ECAP	Obertrubach, Germany	41 (1)	9.62	0.23	< 700 km	
**North & Central America**						
Multiscale Modeling of Astrophysical and Space Plasmas	New York City, USA	41 (1)	57.61	1.41	< 300 km	
2019 Sagan Exoplanet Summer Workshop	Pasadena, California, USA	35 (12)	31.24	0.89	< 100 km	
GROWTH astronomy school	San Diego, USA	149 (62)	102.15	0.69	< 300 km	
CfAO Summer school on adaptive optics	Santa Cruz, California, USA	48 (9)	60.10	1.25	< 300 km	
Summer school on statistics in Astronomy	State College, Pennsylvania, USA	50 (10)	51.38	1.03	< 500 km	
Fermi Summer School	Lewes, Delaware, USA	28 (0)	37.48	1.34	< 300 km	Only country of origin known
**Oceania**						
ASTRO 3D East Coast writing retreat	Sydney, Australia	19 (15)	0.93	0.05	no train	
ASTRO 3D proposal writing workshop	Canberra, Australia	21 (14)	1.13	0.05	no train	Remote attendants excluded
CASS Radio Astronomy School 2019	Narrabri, Australia	57 (1)	33.53	0.59	< 500 km	
**South America**						
First Light	Sao Paulo, Brazil	117 (26)	198.4	1.98	< 500 km	
La plata international school	La Plata, Argentina	57 (24)	54.2	0.95	< 300 km	

Supplementary Table 4: List of conferences lacking data on the origin cities, countries or continents for the participants. If not noted otherwise, the average participant number for each continent was used depending on the conference location. We use the continent-specific mean emissions per participant to estimate the total amount of travel-related GHG emission for each meeting (see Table 1).

Name	Venue	Number of participants	Tot. travel emissions (tCO_2e)	Note
		Asia		
Baryons in Galaxies and Beyond	Teheran, Iran	80	88.0	Participant number estimated from picture
Gamma-Ray Bursts and Related Astrophysics in Multi-Messenger Era	Nanjing, China	91	100.1	
14th International Astronomical Consortium for High Energy Calibration meeting	Kanagawa, Japan	58	63.8	Participant number estimated from programme and picture
Atomic Structure and Oscillator Strengths for astrophysical and fusion plasma research	Shanghai, China	91	100.1	
15th International Symposium on Origin of Matter and Evolution of Galaxies	Kyoto, Japan	91	100.1	
Astrophysical Dynamics	Shanghai, China	91	100.1	
Progenitors of Type Ia supernovae	Lijiang, China	100	110.0	Organisers report ~ 100 participants
Galaxy Angular Momentum Alignment 2019	Shanghai, China	91	100.1	
In the Spirit of Lyot 2019	Tokyo, Japan	91	100.1	
Planet2/RESCEU Symposium: From Protoplanetary Disks through Planetary System Architecture to Planetary Atmospheres and Habitability	Okinawa, Japan	91	100.1	
IRIS 10 @ IIA and CHRIST	Bangalore, India	91	100.1	
		Europe		
Thermal Models in Planetary Science III	Budapest, Hungary	45	45.0	Participant number estimated from programme
Enabling LISA Science Exploitation	Leiden, Netherlands	40	40.0	Participant number based on typical size of Lorentz-Center workshops
MOSAIC 2019: Science and Surveys with the ELT Multi-Object Spectrograph	Heidelberg, Germany	50	50.0	Participant number guessed
UK Exoplanet Community Meeting	London, UK	103	103.0	
Electron-Capture-Initiated Stellar Collapse	Leiden, Netherlands	40	40.0	Participant number based on typical size of Lorentz-Center workshops
Light in the suburbs: structure and chemo-dynamics of galaxy halos	Sexten, Italy	103	103.0	
Supermassive Black Holes: environment and evolution	Corfu Town, Greece	103	103.0	
Stellar Hydro Days V	Exeter, UK	103	103.0	
Feedback and its Role in Galaxy Formation	Spetses, Greece	100	100.0	Organisers report ~ 100 participants
CosmoCruise2019: From the Early to the Late Universe	Venice, Italy	103	103.0	
Understanding the nearby star forming Universe with JWST	Courmayeur, Italy	50	50.0	Participant number estimated from picture
Irish National Astronomy Meeting 2019	Armagh, UK	103	103.0	
The Pleiades and friends: stellar associations in the Gaia era	A Coruña, Spain	103	103.0	
6th Workshop on Robotic Autonomous Observatories	Torremolinos, Spain	103	103.0	
Interaction of Stars with their Environment 2019	Budapest, Hungary	30	30.0	Participant number guessed
PLATO atmospheres workshop	Berlin, Germany	40	40.0	Participant number guessed
Plato Workshop - Single, Shallow, and Strange transits	Warwick, UK	103	103.0	
Exoplanet Vision 2050 workshop	Budapest, Hungary	70	70.0	Max. participants ~ 70
South East Exoplanet Meetings	London, UK	30	30.0	Participant number guessed
		North & Central America		
Midwest Magnetic Fields Workshop 2019	Madison, WI, USA	40	48.0	Participant number estimated from programme
MMT Observatory 40th Anniversary Symposium	Tucson, AZ, USA	110	132.0	
50th Annual Division on Dynamic Astronomy Meeting	Boulder, CO, USA	110	132.0	
Stellar Magnetism: A Celebration of the Contributions of J.D. Landstreet	London, Canada	40	48.0	Participant number estimated from proceedings
Python in Astronomy 2019	Baltimore, MD, USA	50	60.0	Participant number estimated from picture
Understanding Dust 30 Years After CCM	Boulder, CO, USA	50	60.0	Participant number guessed
Astrophysics in the LIGO/Virgo Era	Aspen, CO, USA	20	24.0	Participant number guessed
258th ACS National Meeting, Symposium on Water in the Universe	San Diego, CA, USA	45	54.0	Participant number guessed
SciCoder Workshop	Kampala, HI, USA	20	24.0	Participant number guessed
2019 AAVSO 108th Annual Meeting	Las Cruces, NM, USA	80	96.0	Participant number guessed
NASA Hubble Fellows Program Symposium	Washington DC, USA	24	28.8	Participant number guessed
Brown Dwarf to Exoplanet Connection III	Newark, DE, USA	45	54.0	Participant number guessed from two previous meetings
AO4ELT6 Adaptive Optics for Extremly Large Telescope	Quebec, Canada	210	252.0	Participant number estimated from picture
Emerging Researchers in Exoplanet Science	Ithaca, NY, USA	60	72.0	Participant number guessed
WFIRST Exoplanet Imaging Data Challenge	New York City, NY, USA	20	24.0	Participant number guessed
		Oceania		
ANITA workshop and astroinformatics 2019	Melbourne, Australia	80	26.5	Participant number estimated from picture
ASTRO 3D writing retreat	Coogee, NSW, Australia	19	30.4	Participant number guessed
		South America		
II Workshop Chemical Abundances in Gaseous Nebulae: Open Problems in Nebular Astrophysics	Sao Jose dos Campos, Brazil	60	72.0	Participant number estimated from picture
IAU Symposium 354 - Solar and Stellar Magnetic Fields: Origins and Manifestations	Copiapo, Chile	70	84.0	Participant number estimated from proceedings

Supplementary Table 5: List of schools lacking data on the origin cities, countries or continents for the participants. If not noted otherwise, the average participant number for each continent was used depending on the conference location. We use the continent-specific mean emissions per participant to estimate the total amount of travel-related GHG emissions for each meeting (see Table 1 in the main paper manuscript).

Name	Venue	Number of Participants	Tot. travel emissions (tCO_2e)	Note
		Asia		
IIA Summer Programme	Kodaikanal, India	38	24.70	Number of participants known, assumed mainly Indian origin
Kodaikanal winter school on solar physics	Kodaikanal, India	40	26.00	Maximum number of participants known
NARIT-IOGS Optical Design Summer School 2019	Chiang Mai, Thailand	25	16.25	Used participant numbers from similar school in 2016
”BASIS” Foundation Summer School 2019: Evolution of Galaxies and Stars	Sotchi, Russia	25	16.25	Participant number guessed from programme
Particles and Cosmology: the 16th Baksan School on Astroparticle Physics	Baksan Valley, Kabardino-Balkaria, Russian Federation	25	16.25	Participant number estimated from picture
		Europe		
Ecole Evry Schatzman du PNPS - Interactions étoile - planètes	France	54	37.26	
Computational Fluid Dynamics in Astrophysics	La Laguna, Tenerife, Spain	65	44.85	Participant number estimated from picture
XIII Tonale Winter School on Cosmology	Tonale, Italy	45	31.05	Organisers reported participant numbers and continent of origin
Light Anti-Nuclei as a Probe for New Physics	Netherlands	29	37.26	Participant number listed in meeting report
St Andrews Monte Carlo Radiation Hydrodynamics Summer School	St. Andrews, UK	54	37.26	
4th OBSPM/LAM Summer School Galaxy Formation and Evolution in a Cosmological Context	Spetses, Greece	30	30.70	Maximum number of participants taken from previous school
Advancing Theoretical Astrophysics	Amsterdam, Netherlands	54	37.26	
Exoclimes Simulation Platform (ESP) Inaugural Summer School	Guarda Val, Switzerland	54	37.26	
